# Overhauser-Enhanced MRI of Elastase Activity from In Vitro Human Neutrophil Degranulation

**DOI:** 10.1371/journal.pone.0057946

**Published:** 2013-02-28

**Authors:** Elodie Parzy, Véronique Bouchaud, Philippe Massot, Pierre Voisin, Neha Koonjoo, Damien Moncelet, Jean-Michel Franconi, Eric Thiaudière, Philippe Mellet

**Affiliations:** 1 CRMSB, UMR 5536, University Bordeaux Segalen, CNRS, Bordeaux, France; 2 INSERM, Bordeaux, France; National Research Council, Italy

## Abstract

**Background:**

Magnetic resonance imaging can reveal exquisite anatomical details. However several diseases would benefit from an imaging technique able to specifically detect biochemical alterations. In this context protease activity imaging is one of the most promising areas of research.

**Methodology/Principal Findings:**

We designed an elastase substrate by grafting stable nitroxide free radicals on soluble elastin. This substrate generates a high Overhauser magnetic resonance imaging (OMRI) contrast upon digestion by the target proteases through the modulation of its rotational correlation time. The sensitivity is sufficient to generate contrasted images of the degranulation of neutrophils induced by a calcium ionophore from 2×10^4^ cells per milliliter, well under the physiological neutrophils concentrations.

**Conclusions/Significance:**

These ex-vivo experiments give evidence that OMRI is suitable for imaging elastase activity from neutrophil degranulation. Provided that a fast protease-substrate is used these results open the door to better diagnoses of a number of important pathologies (cystic fibrosis, inflammation, pancreatitis) by OMRI or Electron Paramagnetic Resonance Imaging in vivo. It also provides a long-expected method to monitor anti-protease treatments efficiency and help pharmaceutical research.

## Introduction

Although anatomical imaging methods like X-ray tomography, ultra-sound and MRI can give fine details many pathologies still escape diagnosis. Early transformations of healthy to pathological tissues are better characterized by their biochemistry than by their anatomy. Thus the trend has turned towards the molecular imaging approach to localize such processes. This branch potentially gives access to antigens and receptors targeting or to biochemical activities like the redox status [1], [2] and enzyme activity[3], [4].

Proteolytic activity is an interesting example of enzyme activity suitable for imaging. Protease activity is normally tightly regulated by a large excess of protease inhibitors. To the 500 protease sequences tagged in the human genome correspond about 100 protease inhibitors[5], some of them expressed at high concentration. Consequently the lifetime of an activated protease in normal tissues is very short. However an uncontrolled protease activity is associated with many diseases. In most situations this is the result of local secretion of a complex mixture of mutually activating proteases, each family of protease being able to inactivate the specific inhibitors of another family. Solid tumors, pancreatitis, rheumatoid arthritis, cystic fibrosis and inflammatory diseases of various origins are significant examples associated with a persistent protease/inhibitor imbalance leaving uncontrolled proteolytic activity.

Acute neutrophil-mediated inflammation and cystic fibrosis have in common the role of neutrophils. These cells are the first white blood cells to migrate towards the damaged site. They organize the innate immune response at the early stage of inflammation[6] within minutes to hours. In the particular case of cystic fibrosis the lungs are partly infected with *Pseudomonas aeruginosa*. The bacteria trigger a massive recruitment of neutrophils which try eliminate the bacteria by releasing the content of their granules and vesicles. However *Pseudomonas aeruginosa* colonies are surrounded by a mucus protecting them against neutrophils attack. Thus a chronic inflammation takes place and granules and vesicles content keep accumulating. Neutrophils carry three kinds of granules and one kind of vesicle. Each are able to release up to forty varieties of molecules[7]. Proteases secreted by the azurophil granules and particularly the human neutrophil elastase (HNE) are the most deleterious molecules for the lungs. The term elastase defines a group of enzymes capable of the proteolytic release of soluble peptides from insoluble elastin[8]. Thus during cystic fibrosis elastin of the pulmonary alveoli is fragmented and the lungs progressively lose their function. As a result neutrophil proteases and particularly neutrophil elastase have long been therapeutic targets[9], [10]. However, anti protease treatments have failed so far to significantly improve the status of cystic fibrosis patients[11]. This can be attributed to the absence of any method to monitor the protease activity in vivo and to differentiate the pulmonary regions that are protected by the treatment from those that still undergo elastolytic activity. An imaging method of the elastolytic activity would thus be a great help to evaluate cystic fibrosis patients status and to develop new treatments.

The concept of taking advantage of the proteolytic activity for magnetic resonance imaging (MRI) has been explored by several authors either using gadolinium derivatives [12], [13] or Overhauser magnetic resonance imaging (OMRI) [4]. OMRI is a double resonance experiment establishing a polarization transfer from the free electron of a stable free radical to the surrounding water protons. OMRI seems to be one of the most promising method for protease activity imaging for mainly two reasons. First, it can provide high contrasts and 3D well-resolved images as seen recently on tumor bearing mice [14]. Furthermore the involvement of a free radical is favorable to molecular imaging since the free electron is very sensitive to various changes in its environment: redox status [15], pH, molecular rotational correlation time [4]. In a previous paper we showed that the proteolytic hydrolysis of nitroxide-labeled bovine serum albumin could be followed in vitro by OMRI with the generation of high contrast[4].

In this paper we provide evidence that OMRI has the required sensitivity to follow a simple physiological event, neutrophil degranulation. A nitroxide-labeled elastin sample was used as a substrate for the human neutrophil elastase released upon the provoked degranulation. The induced reduction of the rotational correlation time was monitored by electronic paramagnetic resonance (EPR) spectroscopy and OMRI. The translation of this work towards *in vivo* protease imaging is discussed.

## Results

### Nitroxide-labeled elastin substrate characterization

**Figure 1:**
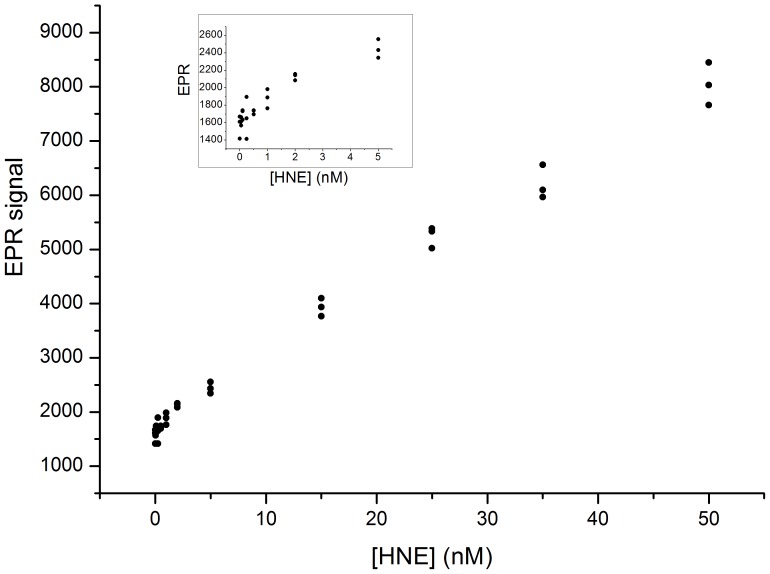
Human neutrophil elastase sensitivity test of nitroxide-labeled elastin substrate probed by EPR. The elastin substrate solution containing 0.36 mM nitroxide was incubated one hour at 37 °C with increasing elastase concentrations. Peak to peak heights of the nitroxide central line versus elastase concentration are plotted. The inset highlights the lowest elastase concentrations.

**Figure 2:**
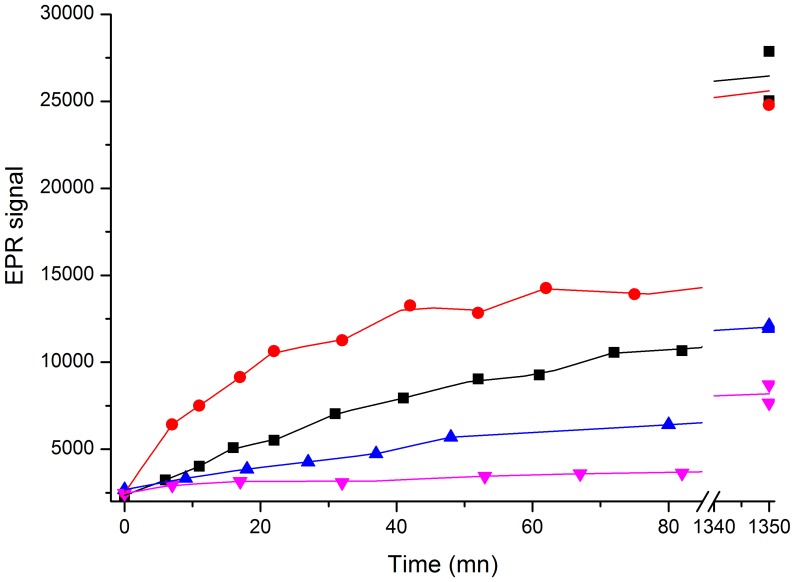
Comparative kinetics of hydrolysis of the elastin substrate by human neutrophil elastase (black squares), porcine pancreatic elastase (red circles), chymotrypsin (blue triangles) and trypsin (pink triangles) probed by EPR. 0.36 mM equivalent nitroxide substrate was incubated at 37°C with 50 nM of each enzyme.

The ability of nitroxide-labeled elastin to generate an EPR signal upon digestion by human neutrophil elastase (HNE) was probed with increasing concentrations of protease. The peak to peak height of the central line of the nitroxide was measured at several incubation times for each concentration. As shown in figure 1, one hour incubation easily discriminates HNE concentrations between 5 and 50 nM. As seen in the inset increasing EPR signal is still detectable in the 0.5 to 5 nM range. Thus EPR detection shows that nitroxide-labeled elastin is a sensitive elastase substrate. The most remarkable property of elastases is their unique capability to release soluble peptides from insoluble elastin[8]. This is due in part to their P1 specificity in the Schechter and Berger nomenclature[16] for valine and alanine which, respectively, constitute 13% and 26% of the amino acid composition of elastin. But the most selective feature is their ability to bind to the fibrous structure of elastin. Since our labeled elastin was made from a solubilized form it was interesting to search whether it has kept some of its elastase substrate specificity. Figure 2 shows the kinetics of hydrolysis by 50 nM human neutrophil elastase, porcine pancreatic elastase, bovine trypsin and chymotrypsin. Nitroxide-labeled elastin retains a good selectivity for both elastases while trypsin is nearly inactive. The slight activity of chymotrypsin is probably due to the chemical denaturation of soluble elastin which unveiled newly accessible cleavage sites.

**Figure 3:**
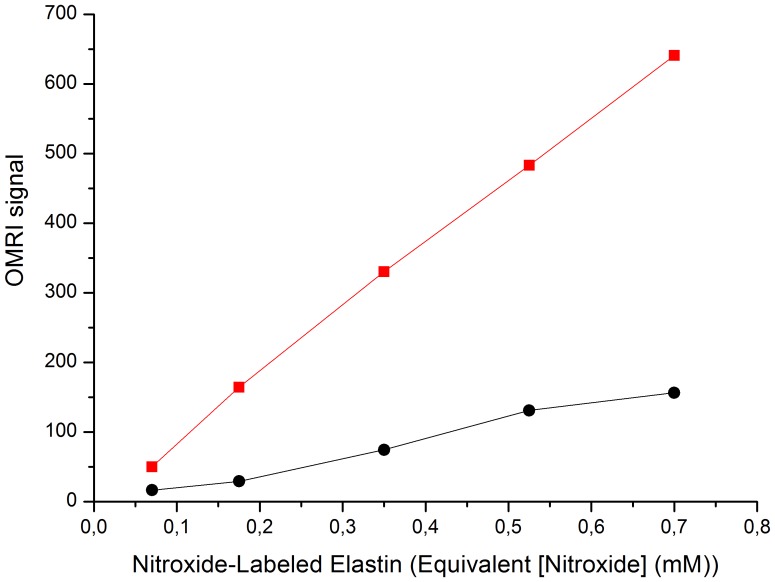
OMRI signal from increasing concentrations of native (black circles) or HNE-digested (red squares) nitroxide-labeled elastin. Complete elastin digestion was carried out during 24 hours with 50 nM HNE at 37°C.

The aim of this work is to generate high contrast images in the presence of elastase activity using Overhauser-enhanced MRI. Various concentrations of nitroxide-labeled elastin, corresponding to a range of 0.07 to 0.7 mM nitroxides, were thus incubated for 24 hours in the presence or absence of human neutrophil elastase. Figure 3 shows that HNE produces a 3 to 5.6 times increase of the NMR signal due to the Overhauser effect for the whole range of concentrations. At 0.07 mM nitroxide concentration the signal is readily 3 times higher in the presence of HNE. Thus nitroxide-labeled elastin is able to generate high contrast images in the presence of HNE at low nitroxide concentration.

### Application to neutrophil degranulation

**Figure 4:**
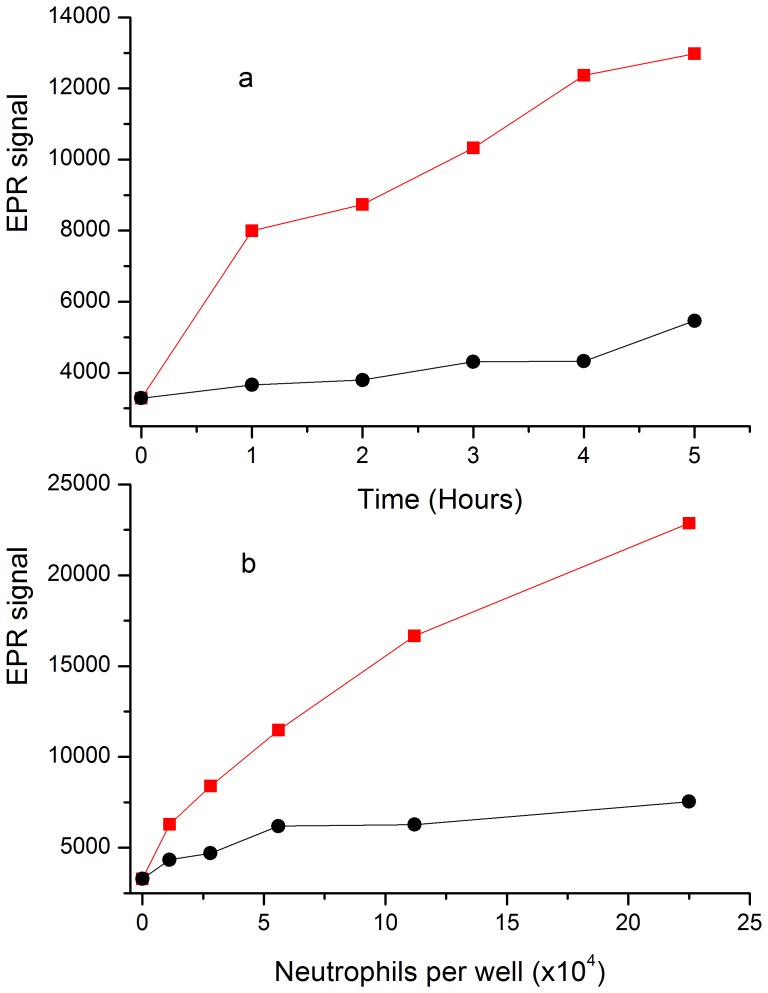
EPR detection of neutrophil degranulation in the presence of nitroxide-labeled elastin (1 mM equivalent nitroxide concentration). (a) EPR signal from 11×10^4^ resting (black circles) or activated (red squares) neutrophils in 0.5 ml versus incubation time at 37°C. (b) EPR signal at five hours incubation at 37°C versus the number of resting (black circles) or activated (red squares) neutrophils.

To mimic a situation of inflammation human neutrophils were purified and incubated with the nitroxide-labeled elastin substrate. Degranulation was induced by adding A23187 calcium ionophore. The generated EPR signal intensities were compared to those from samples without ionophore. In figure 4a the EPR signal intensity clearly discriminates the samples with induced neutrophil degranulation from the samples with resting neutrophils at all times of observation. At each time point a faint signal increase without ionophore could be observed. This can easily be explained by some spontaneous cell death over time which triggers the release of granules. As seen in figure 4b at a given time of observation the EPR signal is strongly correlated to the number of neutrophils per well. Again, in the samples without ionophore spontaneous cell death generates a detectable signal however easily discriminated from the one in induced samples.

**Figure 5:**
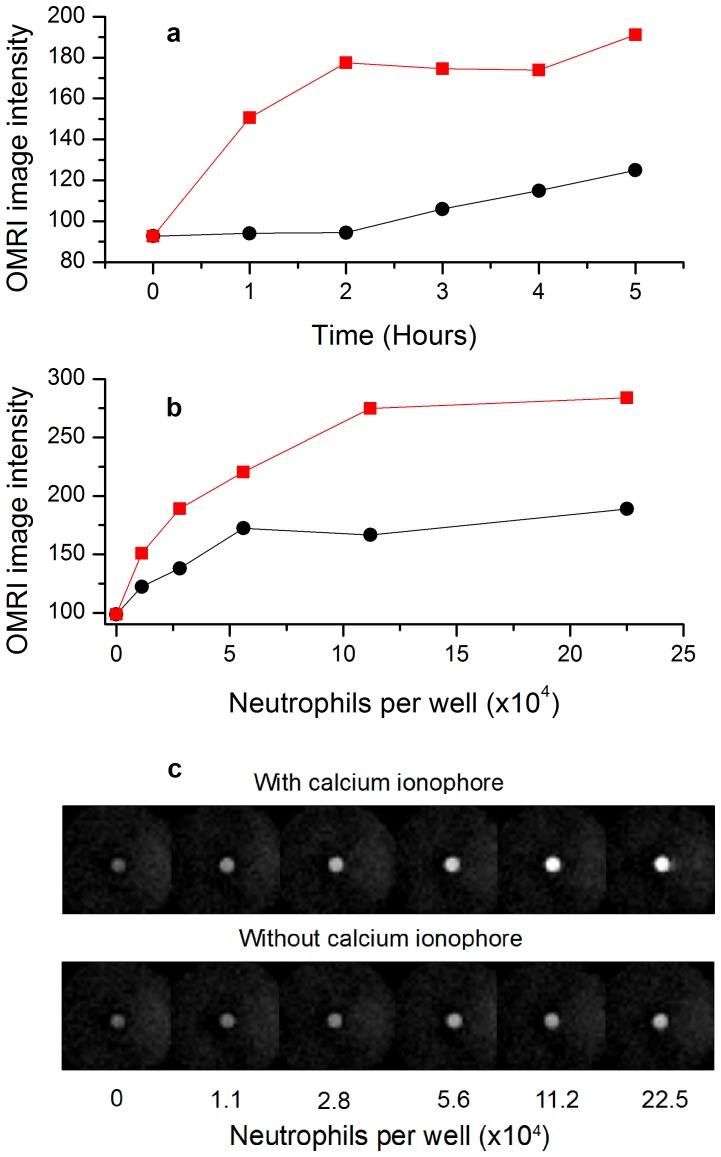
OMRI detection of neutrophil degranulation in the presence of nitroxide-labeled elastin (1 mM equivalent nitroxide concentration). (a) OMRI intensity from 11×10^4^ resting (black circles) or activated (red squares) neutrophils in 0.5 ml versus time of incubation at 37 °C. (b) OMRI intensity at five hours incubation at 37°C versus the number of resting (black circles) or activated (red squares) neutrophils. In (c) the images corresponding to plot (b) are displayed. Gradient Echo (Fast Low Angle SHot) trans-axial images were acquired with the following parameters: TR: 300 ms, TE: 10 ms, RF nutation angle: 70 degrees, Field of View (FOV): 22 mm*22 mm, acquisition matrix: 64*64, in plane resolution: 0.34 mm*0.34 mm, slice thickness: 3 mm, 2 averages. OMRI HF irradiation was applied for 260 ms out of 300 ms TR.

The potential capabilities of this substrate for imaging was probed using OMRI with the same neutrophil containing samples as in the previous EPR study. The kinetics shown in Figure 5a highlights a strong increase of the image intensity in the presence of the calcium ionophore, to be compared with the slow and moderate increase measured in the absence of ionophore. In figure 5b the image intensity versus the number of neutrophils undergoes a significantly steeper increase in the presence of calcium ionophore. In comparison to the EPR signal intensity curves OMRI intensity curves show less linearity at low enzymatic activity points which appear to be overestimated. This feature would be favorable to in vivo OMRI since low proteolytic activities would have a better chance to be detected.

The actual OMRI images used to produce figure 5b are shown in figure 5c. In the presence of ionophore the image intensity enhancement is visible from the first neutrophil concentration (1.12×10^4^ cells/500 µl). Interestingly, the signal enhancement due to elastase release from spontaneous cell death is also visible in the series without ionophore. In these examples of images the intensity enhancement produced by the elastase activity created a contrast equivalent to five times the basal intensity level of control images with nitroxide-labeled elastin but without adding neutrophils. Such an unusually high contrast, unattainable with conventional MRI, should facilitate image interpretation in vivo. Thus in an ex vivo system mimicking a situation of inflammation OMRI is sensitive enough to display high contrast images.

## Discussion

Nitroxide-labeled elastin proves to be a sensitive elastase substrate for both EPR and OMRI. Moreover, this substrate was applied to detect a nearly physiological event, namely human neutrophils degranulation ex vivo, by EPR spectroscopy and to produce images of this event by OMRI. Significantly, the concentrations of cells in this experiment, 2.2×10^4^ to 45×10^4^ cells/ml, were well under the neutrophil concentration in normal blood (2×10^6^ to 7.5×10^6^ cells/ml). The target of our substrate, human neutrophil elastase, is an important enzyme in several pathologies. Chronic obstructive pulmonary disease[17], rheumatoid arthritis[18], atherosclerosis[19] or cystic fibrosis all involve HNE activity at some stage of the disease. For instance in the pulmonary purulent sputum of the cystic fibrosis patients up to 5.10^−6^ M active human neutrophil elastase has been found[11]. It is worth to notice that in the present work the sensitivity for active HNE was in the nanomolar range with EPR spectroscopy and OMRI imaging. Neutrophils are the first cells from the innate immune system to migrate towards an injured or infected site. For instance bronchoalveolar lavage fluid from young children with cystic fibrosis may contain tens of million cells per ml[20]. Thus neutrophil elastase activity is a good marker of any kind of acute or chronic inflammation and an interesting target for imaging. Therefore the proposed imaging method based on elastolytic activity would favor progresses in basic research, diagnosis and treatment follow up.

The activity from pancreatic elastase can also be spotted by our method (see fig. 2). In pancreatitis this elastase is prematurely activated in the pancreas instead of being secreted as an inactive zymogen which is then activated in the duodenum. Pancreatitis can appear as a chronic or acute form and its etiology can either be gallstones, fat diet, alcohol or inherited zymogen defects. Understanding and evaluating the evolution of the disease during therapy, *e.g.* anti-protease treatments[21], would be made easier with a non-invasive whole-body protease imaging method, particularly in the early stages of chronic pancreatitis[22].

Mouse OMRI at 0.2 Tesla has proven to be an efficient method to follow nitroxide biodistribution in 3D at high resolution[14]. Thus ex vivo elastase imaging carried out with the same OMRI system opens the way to in vivo inflammation imaging, where protease activity is high enough to overcome the protection provided by protease inhibitors. To actually observe significant signal enhancement in vivo the free nitroxide concentration should remain in the range of 0.1 to 1 mM over several minutes. This requires both a good biodistribution of the substrate and a fast hydrolysis of the substrate by the enzyme to compensate for the diffusion of the free nitroxide and its renal clearance. In this paper we used a natural protein which undergoes a typical slow hydrolysis by the elastases thus limiting the chances for useful in vivo applications. It is however possible to use small nitroxide containing peptides including an elastase specific cleavage site. These peptides would then be linked to a protein or nanoparticle carrier to lower their rotational diffusion coefficient. The nitroxide would then be released from the carrier by the target enzyme hence providing OMRI contrast. In this way the kinetics of hydrolysis can be raised by several orders of magnitude.

Incidentally, nitroxide-labeled elastin is an excellent substrate to quantify elastase activity by EPR in opaque media where optical methods fail. Such substrates might consequently be used in EPR imaging (EPRI) of protease activity. However significant developments are needed to provide actual 3D EPR images at sub-millimeter spatial resolution within several minutes.

## Materials and Methods

### Enzymes

Human neutrophil elastase and porcine pancreatic elastase were purchased from Elastin Products company (Missouri, USA). Bovine trypsin and chymotrypsin were from Worthington (New Jersey, USA).

All experiments involving purified proteases were done in 0.1 M phosphate buffer pH 7.4 at 37°C.

### Nitroxide-labeled elastin

Elastin-soluble (Elastin Products Company, Missouri, USA) is bovine neck ligament elastin extracted and processed with hot oxalic acid. The crude product is thus a mixture of peptides ranging from 3 kDa to 60000 kDa. In this paper the Overhauser switch relies on the initial high rotational correlation time of the substrate. Thus an initial molecular weight selection was done by centrifuging three times a 15 ml solution of 500 mg of elastin in 20 mM phosphate buffer at pH 7.2 on concentrating filters with a cut-off of 30000 kDa (Amicon Ultra 30 K, Millipore). The retained molecules were diluted in 15 ml of 20 mM phophate buffer pH 8.3 and incubated overnight with 50 mg 1-Oxyl-2,2,5,5-tetramethylpyrroline-3-carboxylate N-Hydroxysuccinimide Ester (Toronto Research Chemicals, Canada). The mixture was then concentrated to 2 ml with the same type of filter. A second size selection was done by gel filtration through a Biosuite 250 21.5×300 mm HPLC column (Waters) at 4 ml/mn in 0.1 M phosphate buffer with 0.15 M NaCl at pH 7.4. Fractions up to 20 minutes were collected and concentrated to 3.5 ml.

**Figure 6:**
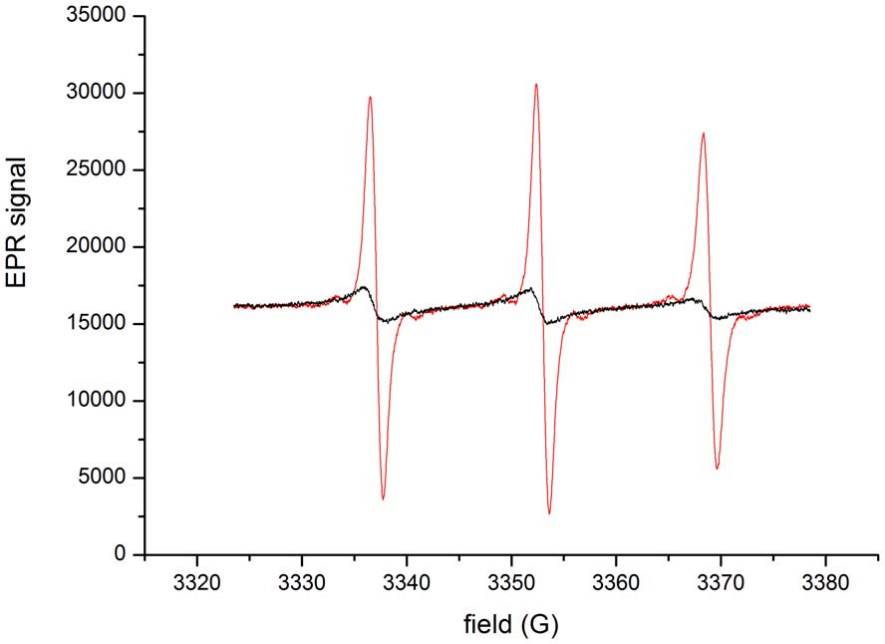
Typical EPR spectra of native (black line) and HNE digested (red line) nitroxide-labeled elastin (0.36 mM equivalent nitroxide).

Characterization of nitroxide-labeled elastin: Figure 6 shows the EPR spectra of elastin before and after complete proteolysis with Human Neutrophil Elastase.

The concentration of nitroxide in the stock solution (18 mM) was measured by integrating the central line of the digested sample and comparing with a proxyl calibration line (not shown).

### Neutrophil isolation and characterization

**Figure 7:**
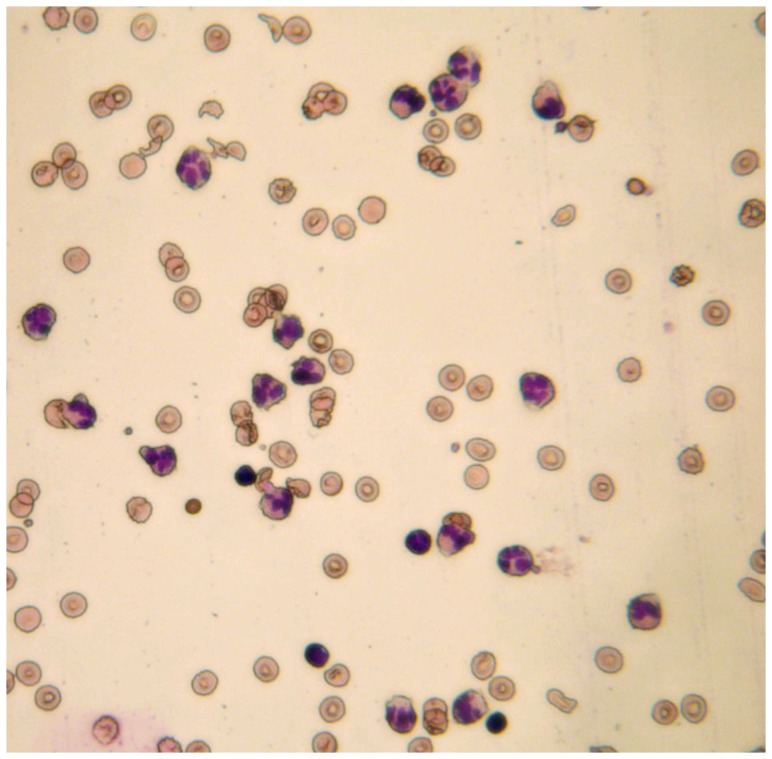
Representative optical microscope field from the May-Grünwald-Giemsa staining of the neutrophil-enriched preparation used for the degranulation experiments.

Neutrophils were isolated from a leukoreduction filter kindly provided by the French Blood Service (Bordeaux, France). The filter was back-flushed with 50 ml of DMEM (gibco) completed with BSA (40 g/l)(Sigma), Citrate-dextrose solution (10% vol/vol) (Sigma) and Pulmozyme, a dornase alpha commercial solution (10 µl/ml) at pH 7.4. The cells were diluted to 160 ml with the same solution and spun 20 mn at 110 g and 20°C in four tubes. The pellets were re-suspended in 90 ml of DMEM with dornase, layered on six tubes containing 15 ml of Granulosep (Eurobio) and spun 20 minutes at 1500 g and 20°C. The interface containing the white cells was harvested and washed in two times 50 ml of DMEM with dornase. Each pellet was resuspended in 20 ml DMEM with dornase, layered on 10 ml of Lymphocyte Separation Medium (Eurobio) and spun 40 mn at 400 g and 20°C. The pellet was highly enriched in granulocytes but still contained red cells and a few lymphocytes as seen in figure 7. It was harvested and washed with DMEM and was used as such in further experiments since neither red cells nor lymphocytes are able to release elastase. Neutrophils counting was carried out from a sample diluted in red cells lysing solution (Becton Dickinson) washed in phosphate buffer saline solution and analyzed on a Guava easyCyte flow cytometer/counter (Millipore). The neutrophil population was identified and counted according to its forward scatter versus side scatter properties.

In all experiments neutrophils were incubated in DMEM without serum at 37 °C in 5% CO_2_ atmosphere. Neutrophil degranulation was induced with 2.5 10^−6^ M calcium ionophore A23187 (Sigma). At the end of the incubation time eglin c, a rapid high affinity elastase inhibitor[23] was added in excess before sample freezing.

### Electronic Paramagnetic Resonance

All EPR spectra were recorded with a MiniScope MS200 EPR spectrometer (Magnettech, Berlin, Germany). B0-field was set to 3350 G, sweep range to 45 G in 60 seconds, modulation to 100 mG. The gain was constant inside each series of spectra. The samples were loaded in 75 mm/60 µl capillaries (Hirschmann Laborgerate, Germany).

### Dynamic Nuclear Polarization enhanced Magnetic Resonance Imaging

#### EPR Cavity and MRI devices

A C-shaped MRI system, Magnetom Open Viva operating at 0.194T (Siemens, Erlangen, Germany) was used. The proton frequency was 8.24 MHz. Gradients maximum intensity was 15 mT m^−1^. Electron spin excitation was induced by a resonant hyperfrequency (HF) cylindrical cavity (Bruker, Wissembourg, France) running in TE011 mode, positioned at the center of the MRI magnet bore [4]. Its geometry (240 mm diameter, 29 mm width) was designed to reduce the electric component of the HF Field in its center, thus minimizing sample heating upon microwave emission. Actual power deposition was evaluated by measuring temperature elevation with a temperature probe placed in a phosphate-buffered saline phantom (25 mm in diameter). By neglecting conduction, a heat equation was used to fit the initial linear temperature changes upon microwave excitation. Peak power was estimated to be in the range of 4 W. Previous in vivo experiments in the same conditions were harmless to mice[14]. The usable magnetic component was concentrated at the center of this cavity where an opening from both sides allowed sample access. The sample area at the center of the cavity was 28 mm in diameter and 29 mm in length. The HF amplification channel and the cavity were fully described in a previous paper[4]. Modification for minimizing the Eddy currents in order to improve MRI image quality was described previously[14].

Each sample was loaded into a 1.6 mm internal diameter capillary mechanically sealed at both ends. Each capillary was then positioned at the center of a 12 mm plastic tube, filled with water in order to load the MRI coil.

#### Pulse sequences

Two-dimensional magnetic resonance images were obtained with a standard Gradient Echo sequence. MRI acquisition and HF pulses were synchronized by an external pulse generator (RFPA, Artigues-pres-Bordeaux, France), allowing modulation of HF pulse duration. The HF pulse duration was 260 ms to be compatible with the T_1_ value of water in the presence of nitroxide in the millimolar range. It was immediately followed by the MRI sequence with an echo time (TE) of 10 ms and minimal TR of 27 ms. This scheme was repeated for each echo acquisition. The effective TR, including HF irradiation time, was then 300 ms. All MR adjustments were done manually, using the same fixed receiver amplification gain for both measurements, without and with HF irradiation, so that signals can be directly compared and Overhauser enhancements calculated.

#### Post processing

Post-processing evaluations were carried out with IGOR Pro (Wavemetrics, Lake-Oswego, OR, USA). All signal intensity measurements were done with ImageJ imaging software (ImageJ, National Institutes of Health, USA). Signal intensity was measured in a circular region of interest of 1 mm^2^ positioned in the capillary area.
