# Correction: Overhauser-Enhanced MRI of Elastase Activity from In Vitro Human Neutrophil Degranulation

**DOI:** 10.1371/annotation/47f8c256-07a4-44a1-88cb-8c461e0d45e4

**Published:** 2013-08-13

**Authors:** Elodie Parzy, Véronique Bouchaud, Philippe Massot, Pierre Voisin, Neha Koonjoo, Damien Moncelet, Jean-Michel Franconi, Eric Thiaudière, Philippe Mellet

A funding organization and grant for the fifth author were incorrectly omitted from the Funding Statement. The following sentence should be read as the second sentence of the Funding Statement: "Neha Koonjoo was supported by TRAILDNP from the public grant of the French 'Agence Nationale de la Recherche' within the context of the Investments for the Future Program, referenced ANR-10-LABX-57 and named TRAIL." 

